# The obesity paradox in primary Sjögren’s disease: immunometabolic associations of chemerin, platelet count, and statin use

**DOI:** 10.3389/fimmu.2026.1890786

**Published:** 2026-09-21

**Authors:** Kincső Mezei, Viktória Orosz, Ildikó Fanny Horváth, Zsófia Aradi, Hajnalka Lőrincz, Mariann Harangi, Antónia Szántó

**Affiliations:** 1 Division of Clinical Immunology, Institute of Internal Medicine, Faculty of Medicine, University of Debrecen, Debrecen, Hungary; 2 Gyula Petrányi Clinical Immunology and Allergology Doctoral School, University of Debrecen, Debrecen, Hungary; 3 Division of Metabolism, Department of Internal Medicine, Faculty of Medicine, University of Debrecen, Debrecen, Hungary; 4 Institute of Health Studies, Faculty of Health Sciences, University of Debrecen, Debrecen, Hungary; 5 Eötvös Loránd Research Network-University of Debrecen (ELKH-UD) Vascular Pathophysiology Research Group, University of Debrecen, Debrecen, Hungary

**Keywords:** adipokine, chemerin, obesity, oxLDL, Sjögren’s disease, statin

## Abstract

**Introduction:**

Sjögren’s disease is characterized by autoimmune destruction of exocrine glands. Obesity generally worsens the course of many diseases; however, earlier observations in our cohort suggested a paradoxically lower disease activity (ESSDAI) in overweight/obese patients. This cross-sectional study aimed to investigate potential mechanisms underlying these findings.

**Methods:**

Chemerin and oxidized low-density lipoprotein (oxLDL) levels were measured in a subset of regularly followed patients (n=75). Patients were stratified by body mass index (BMI): BMI <25 kg/m² (“non-obese”, n=27) and BMI ≥25 kg/m² (“overweight/obese”, n=48). Demographic, laboratory, and immunological parameters, disease activity (ESSDAI), extraglandular manifestations, and treatments were compared using biostatistical analyses.

**Results:**

Chemerin and oxLDL levels did not differ significantly between groups. ESSDAI was significantly lower in overweight/obese patients (5.04 vs. 2.76; p=0.04), while complement c3 levels were higher (1.20 vs. 1.46 g/L; p<0.001). Across all patients, chemerin positively correlated with BMI, age at diagnosis, c3, c4, CRP, platelet count, and leukocyte count. Hemoglobin and hematocrit were higher in overweight/obese patients, whereas neutrophil ratio was lower and lymphocyte ratio slightly higher. Statin therapy was also more frequent in the overweight/obese group (p=0.04). In this group, BMI and platelet count independently predicted chemerin levels, while platelet count predicted chemerin in normal-weight patients.

**Conclusion:**

Despite its positive correlation with BMI, chemerin levels were not significantly higher in overweight/obese patients, suggesting relatively lower levels. This may partly explain their more favorable immunological profile. Increased statin use may also contribute through anti-inflammatory effects.

## 1 Introduction

Sjögren’s disease is characterized by the autoimmune-mediated destruction of ductal epithelial cells, which leads to various symptoms. Most of them are related to the dysfunction of exocrine glands, such as xerostomia, xerophthalmia, pancreas dysfunction, skin dryness, vaginitis sicca, etc. Besides, it can also cause numerous extraglandular manifestations, for instance, non-erosive polyarthritis, central and peripheral nervous system manifestations, interstitial lung disease, muscular involvement, nephropathy, vasculitis, and different types of autoimmune cytopenia among others (1–3). It is also important to mention accelerated atherosclerosis, which is proved to be a serious complication in autoimmune diseases, researched mainly in systemic lupus erythematosus and rheumatoid arthritis, but it is present in Sjögren’s disease as well, further aggravating its cardiovascular risk (4–7).

Obesity is known to influence a wide range of diseases and is a basic feature of the assessment of risk factors (8, 9), exceeding even tobacco use at this point (10). In our prior paper, we observed that Sjögren’s patients with a normal body weight have higher disease activity compared to obese ones (11). Subsequently, extensive literature research was conducted to unravel the potential causes behind these findings. Three key contributing factors can be assumed. First of all, the obesity paradox is a well-studied phenomenon, which states that metabolically healthy obese individuals have better survival rates in numerous diseases than patients with a normal weight who otherwise live an unhealthy lifestyle (12–14). Secondly, the effect of statin therapy must be mentioned, although in this paper we are not focusing on that, since it was revised in our previous study. The majority of our overweight/obese patients were undergoing contemporary statin treatment, while such medication was used by only a small percentage of the non-obese group. Statins are well recognized for their pleiotropic anti-inflammatory effect, which may ameliorate the clinical course of Sjögren’s disease. By suppressing certain inflammatory pathways and inflammasome activation, they mitigate the risk of endothelial dysfunction (15–18). The effects of these drugs have been studied in autoimmune diseases with promising results, including rheumatoid arthritis, systemic lupus erythematosus, antiphospholipid syndrome and vasculitis (19–21). The third suspected cause behind our results is the role of certain adipokines which provided the idea for our current research.

Adipokines are secreted by the adipose tissue, which essentially acts like an endocrine organ, meaning that these molecules are biologically active and influence the operation of several organs. When a patient is severely obese, the endocrine function of the adipose tissue shifts to an adverse way secreting more pro-inflammatory adipokines compared to anti-inflammatory ones. This leads to the aggravation of local and systemic inflammation, which contributes to the progression of several diseases linked to obesity and also interferes with autoimmune mechanisms (22–25). Our study focused on chemerin and oxidized low-density lipoproteins (oxLDL).

Chemerin is an adipokine which serum levels correlate positively with body mass index (BMI). It plays an important role in inflammatory processes, and its receptors, including chemokin-like receptor-1 (CMKLR1), G-protein coupled receptor-1 (GPR1) and C-C-motif chemokine like receptor (CCRL2), are expressed by adipocytes and inflammatory cells. Chemerin contributes to immune-cell recruitment and immune homeostasis, particularly through its effects on innate immune cells such as dendritic cells and macrophages. It has also been implicated in atherogenesis and metabolic regulation, including glucose metabolism, insulin sensitivity, adipogenesis, and angiogenesis (26–29). More broadly, emerging evidence suggests that obesity-associated inflammation involves complex communication between metabolic and immune organs rather than adipose tissue acting in isolation. The liver–spleen axis has recently been described as an important immunometabolic pathway, in which splenic immune-cell activation and monocyte trafficking through the portal circulation can influence hepatic inflammation and fibrogenesis, while hepatic signals can reciprocally modulate systemic and splenic immune activity (30). This concept further supports the potential relevance of adipokines and other circulating inflammatory mediators in systemic immune-metabolic regulation. In the present study, we therefore focused on chemerin and oxidized low-density lipoproteins (oxLDL).

Oxidized low density lipoproteins are known to modulate inflammatory pathways in several ways, contributing to atherogenesis, influencing the operation of macrophages, plaque formation and plaque instability as well. They also have antigenic potential, through pattern recognition receptors (PRRs), they can secrete cytokines and can bind to specific antibodies forming immune complexes activating inflammatory cells. Therefore, the role of oxLDL is mainly considered disadvantageous, however, recent studies suggest that it may serve as a potential therapeutic target for the prevention of thromboembolism, although more research is needed in that area (31–35).

In this present study, we aimed to further investigate the etiology of lower disease activity of overweight/obese patients. Therefore, we researched whether obesity-associated immune-metabolic factors explain this phenomenon and explored immune-metabolic heterogeneity in Sjögren’s disease.

## 2 Materials and methods

### 2.1 Patient enrolment

Out of our regularly followed patients, chemerin and oxLDL levels were assessed in a subset of 75 people. All patients gave informed consent to participate. Patients were stratified into two groups based on body mass index (BMI): those with a BMI<25 kg/m^2^ were classified as ‘non-obese’ (n=27), while those with a BMI≥25 kg/m^2^ were categorized as ‘overweight/obese’ (n=48). In the non-obese group, the median age was 61, while in the overweight/obese group it was 60. All the patients were women. Demographic parameters (gender, age at diagnosis of Sjögren’s disease), complete blood count, kidney and liver function tests, erythrocyte sedimentation rate (ESR), C-reactive protein (CRP), triglyceride, cholesterol levels, immunological parameters (anti-SS-A, anti-SS-B, complement levels), EULAR Sjögren’s syndrome disease activity index (ESSDAI), certain extraglandular manifestations and associated autoimmune conditions (arthropathy, nephropathy, skin and lung manifestations, cryoglobulinemia, vasculitis, systemic lupus erythematosus, rheumatoid arthritis, antiphospholipid syndrome, autoimmune thyroiditis, Raynaud syndrome) and treatment modalities were compared.

### 2.2 Measurement of routine laboratory parameters

Peripheral blood samples were collected into Vacuette^®^ tubes (Greiner Bio-One Hungary Kft., Mosonmagyaróvár, Hungary). After a half hour rest, tubes were centrifuged at 3500 g 15 min 4 °C and serum/plasma samples were collected. Routine laboratory parameters were measured immediately from fresh samples, while a 500 μl serum aliquot from each patient was stored at -80 °C for subsequent chemerin and oxLDL measurements.

Routine laboratory parameters, including aspartate transaminase (AST), alanine transaminase (ALT), gamma glutamyl transferase (GGT), alkaline phosphatase (AP), lactate dehydrogenase (LDH), estimated glomerular filtration rate (eGFR), urea, creatinine, hematological parameters (leukocyte count, proportion of lymphocytes, neutrophil granulocytes, hemoglobin levels, platelet counts), triglyceride, total cholesterol, low-density lipoprotein-cholesterol [LDL-C], high-density lipoprotein-cholesterol [HDL-C]) were performed according to the standard laboratory methods with a Cobas c600 autoanalyzer (Roche Ltd., Mannheim, Germany) at the Department of Laboratory Medicine, Faculty of Medicine, University of Debrecen, Hungary. Erythrocyte sedimentation rate (ESR), and immune serological markers (presence of anti-Ro/SS-A, anti-La/SS-B, rheumatoid factor, serum immunoglobulin (Ig)A, IgG, IgM and complement levels (c3, c4, 50% hemolytic complement activity (CH50)) were also assessed. All reagents were purchased from the same vendor.

### 2.3 Measurement of chemerin

Serum chemerin concentrations were measured by a commercially available enzyme linked immunosorbent assay (ELISA) kit (Human Chemerin Quantikine ELISA; Cat.No: DCHM00; R&D Systems, Abingdon, UK), according to the recommendations of the manufacturer. The intra- and inter assay variation coefficients were 2.8-4.5% and 6.4-7.9%, respectively. Samples were used in 100-fold dilution and values were expressed as ng/mL.

### 2.4 Measurement of oxLDL

Serum concentrations of oxLDL were measured using ELISA (Mercodia AB, Uppsala, Sweden; Cat.No. 10-1143-01). This assay utilizes a direct sandwich technique where two monoclonal antibodies target distinct antigenic determinants on the oxidized apolipoprotein B molecule. The intra-assay and inter-assay CVs were 5.5-7.3% and 4.0-6.2%, respectively. Samples were used in 6561-fold dilution and values were expressed as U/L.

### 2.5 Statistical analysis

Statistical analyses were performed using the Statistica 13.5.0.17 software (TIBCO Software Inc., Tulsa, OK, USA). GraphPad Prism 8.01 (GraphPad Prism Software Inc., San Diego, CA, USA) was applied to create graphs in the text. Normality of continuous data was tested with the Kolmogorov–Smirnov test. Data were expressed as means ± SD or medians (interquartile range). Chi-square test was used to analyze categorical data by comparing observed frequencies with expected frequencies. Pearson correlation was used to investigate the relationship between selected variables. Backward stepwise multiple regression analyses were performed to determine significant variables predicted chemerin in overall (Model 1), normal-weight (Model 2) and overweight/obese Sjögren’s population (Model 3), respectively. Models were adjusted for BMI, gender, age at the time of Sjögren’s diagnosis, statin use and included all variables that correlated with chemerin in the univariate analyses, i.e. c3, c4, log10hsCRP, oxLDL, platelet count and leukocyte count. Variance inflation factors (VIFs) were calculated in each model to characterize collinearity among the predictors. All VIF values were <5, indicating that multicollinearity was not a concern. P ≤ 0.05 probability values were considered statistically significant.

## 3 Results

### 3.1 Chemerin, oxLDL and ESSDAI

Considering chemerin and oxLDL levels in the non-obese and overweight/obese groups, respectively, there was no significant difference between the two subsets. However, ESSDAI was significantly lower in overweight/obese. (**Table 1**) No significant difference was found between patients with and without statin therapy regarding chemerin and oxLDL levels.

**Table 1:** Differences in body mass index, chemerin and oxLDL levels and EULAR disease activity index (ESSDAI) between the two groups (1: non-obese, 2: overweight/obese).

Variables	Non-obese (mean ± SD)	Overweight/ obese (mean ± SD)	F-ratio variances	p
**BMI**	22.41 ± 2,24	31.06 ± 4,40	3.86	**<0.0001**
Chemerin (ng/ml)	96.43 ± 20.10	106.03 ± 25.88	1.66	0.17
oxLDL (U/L)	98.03 ± 31.23	107.81 ± 37.99	1.48	0.29
**ESSDAI**	5.04 ± 4.26	2.76 ± 2.92	2.13	**0.04**

Bold values indicate statistically significant results in the tables.

### 3.2 Immunological parameters

Regarding immunological parameters, there was no remarkable difference in complement activity (CH50), aSS-A, aSS-B positivity, although c3 and c4 levels were significantly higher in the overweight/obese group. Additionally, the neutrophil granulocyte ratio of overweight/obese patients was significantly lower than in the non-obese group. Regarding immunoglobulins (IgA, IgG, IgM), there was no significant difference between the two groups. (**Table 2**).

**Table 2:** Relevant immunological parameters of the two groups (1: non-obese, 2: overweight/obese).

Variables	Non-obese (mean ± SD)	Overweight/ obese (mean ± SD)	F-ratio variances	p
Lymphocyte ratio (%)	21.86 ± 9.72	29.06 ± 9.26	1.10	0.75
**Neutrophil ratio** **(%)**	**66.95 ± 11.64**	**58.59 ± 10.43**	**1.25**	**0.002**
**c3 (g/)**	**1.20 ± 0.19**	**1.46 ± 0.52**	**6.96**	**<0.001**
**c4 (g/l)**	**0.20 ± 0.08**	**0.27 ± 0.09**	**1.57**	**0.003**
CH50	68.34 ± 18.58	70.67 ± 14.66	1.60	0.16
aSS-A positivity	0.63 ± 0.49	0.48 ± 0.50	1.05	0.21
aSS-B positivity	0.48 ± 0.51	0.29 ± 0.46	1.21	0.12
IgA (g/)	2.31 ± 1.33	2.29 ± 1.40	1.10	0.97
IgG (g/l)	12.86 ± 7.25	10.66 ± 6.76	1.15	0.19
IgM (g/)	1.99 ± 2.67	2.10 ± 3.28	1.49	0.88

Bold values indicate statistically significant results in the tables.

### 3.3 Routine laboratory parameters and medications

As for liver function tests, gamma-glutamyl transferase (GGT and alanine transaminase (ALT)) levels of non-obese patients were significantly lower. Hemoglobin and hematocrit were significantly higher in overweight/obese patients. Cholesterol levels of the overweight/obese group were lower; however, triglyceride levels were significantly higher (**Table 3**). Also, not surprisingly, in the overweight/obese category, statin therapy was administered significantly more frequently (18,52% vs. 41,67%; p=0.04).

**Table 3:** Relevant routine laboratory parameters of the two groups (1: non-obese, 2: overweight/obese).

Variables	Non-obese (mean ± SD)	Overweight/ obese (mean ± SD)	Std. dev. 1	Std. dev. 2	F-ratio variances	p
**GGT** **(U/L)**	**26.70 ± 25.26**	**48.23 ± 58.23**	**25.26**	**58.23**	**5.32**	**<0.001**
**ALT** **(U/L)**	**21.67 ± 17.82**	**29.13 ± 31.78**	**17.82**	**31.78**	**3.18**	**0.002**
**Hemoglobin** **(g/)**	**126.15 ± 14.69**	**133.48 ± 10.59**	**14.69**	**10.59**	**1.92**	**0.014**
**Hematocrit**	**0.38 ± 0.042**	**0.39 ± 0.03**	**0.042**	**0.03**	**2.38**	**0.01**
Platelet count (G/L)	263.74 ± 75.33	244.69 ± 64.88	75.33	64.88	1.35	0,25
Cholesterol (mmol/L)	5.05 ± 1.22	4.95 ± 1.24	1.22	1.24	1.03	0.73
**Triglyceride** **(mmol/L)**	**1.15 ± 0.48**	**1.58 ± 0.65**	**0.48**	**0.65**	**1.81**	**0.004**

Bold values indicate statistically significant results in the tables.

### 3.4 Correlations with chemerin and oxLDL levels

Since comparative tests did not reveal considerable results concerning adipokines, we also examined the correlation of certain parameters with chemerin and oxLDL levels of all our patients, without categorizing them. A significant positive correlation was found between serum chemerin level and BMI (**Figure 1A**), age at diagnosis (**Figure 1B**), c3 (**Figure 2A**), c4 (**Figure 2B**), C-reactive Protein (CRP) (**Figure 2C**), platelet count (**Figure 3A**) and leukocyte count (**Figure 3B**). Examining the correlation of ESSDAI, a negative correlation was found with serum chemerin levels, however, it was not significant (r=-0.073, p=0.578). In case of oxLDL, we found significant positive correlations with cholesterol levels (r=0.626, p<0.001), and with leukocyte count (r=0.276, p=0.017). Regarding further laboratory parameters (e.g. kidney function, liver enzymes, autoantibodies), correlation results were not significant.

**Figure 1:**
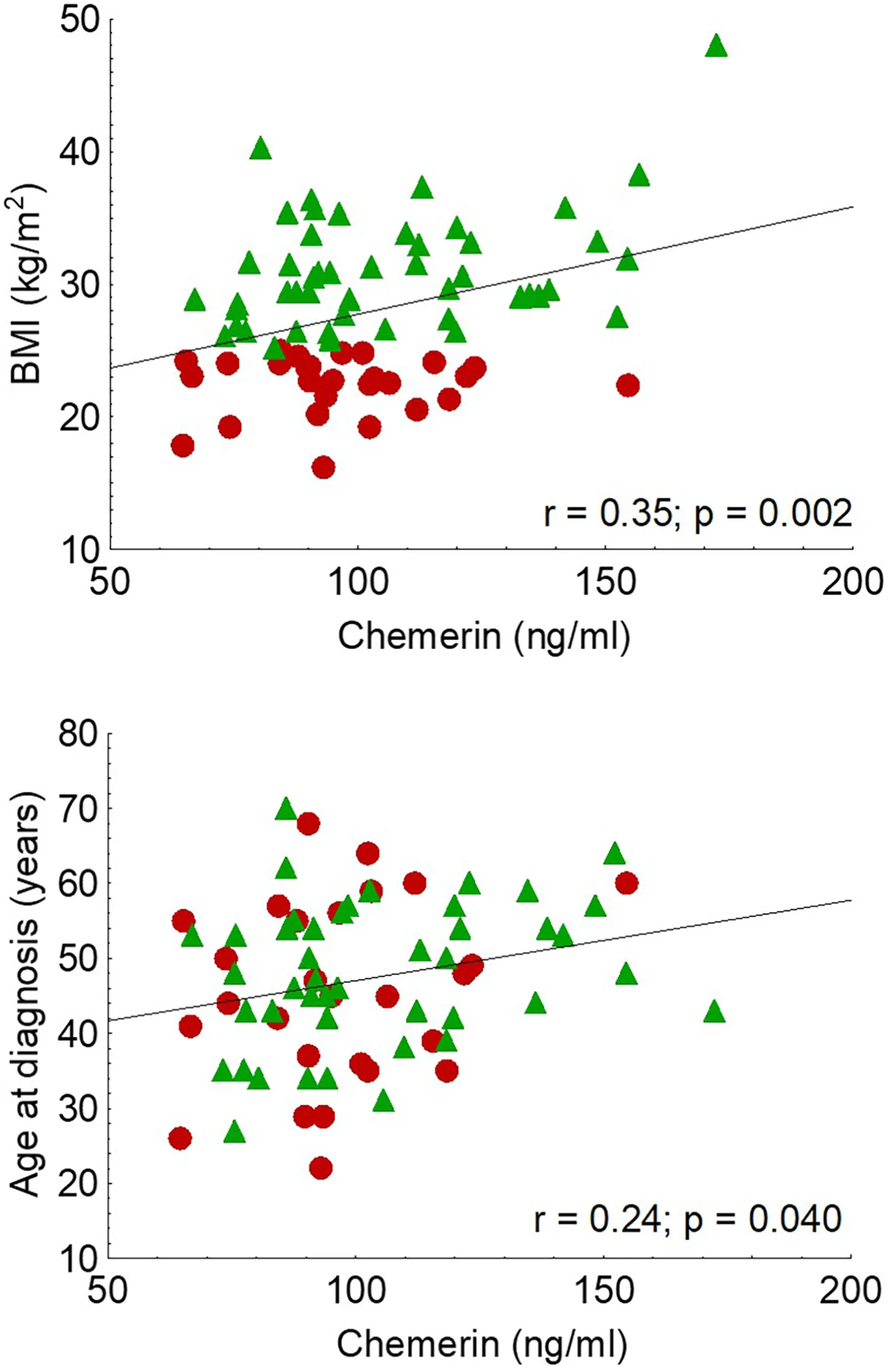
Correlation between BMI and chemerin levels (green: overweight/obese, red: non-obese). Correlation between age at diagnosis and chemerin levels (green: overweight/obese, red: non-obese).

**Figure 2:**
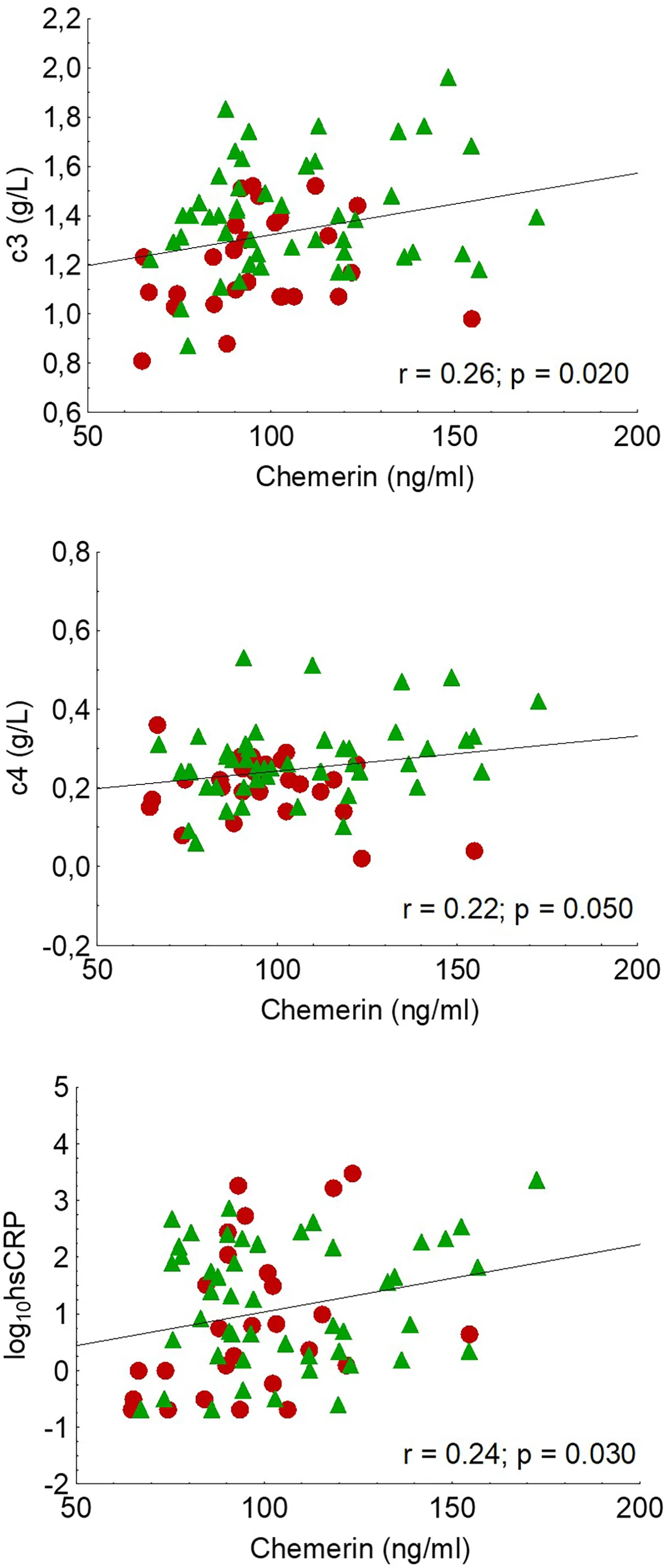
Correlation between c3 and chemerin levels (green: overweight/obese, red: non-obese). Correlation between c4 and chemerin levels (green: overweight/obese, red: non-obese). Correlation between CRP and chemerin levels (green: overweight/obese, red: non-obese).

**Figure 3:**
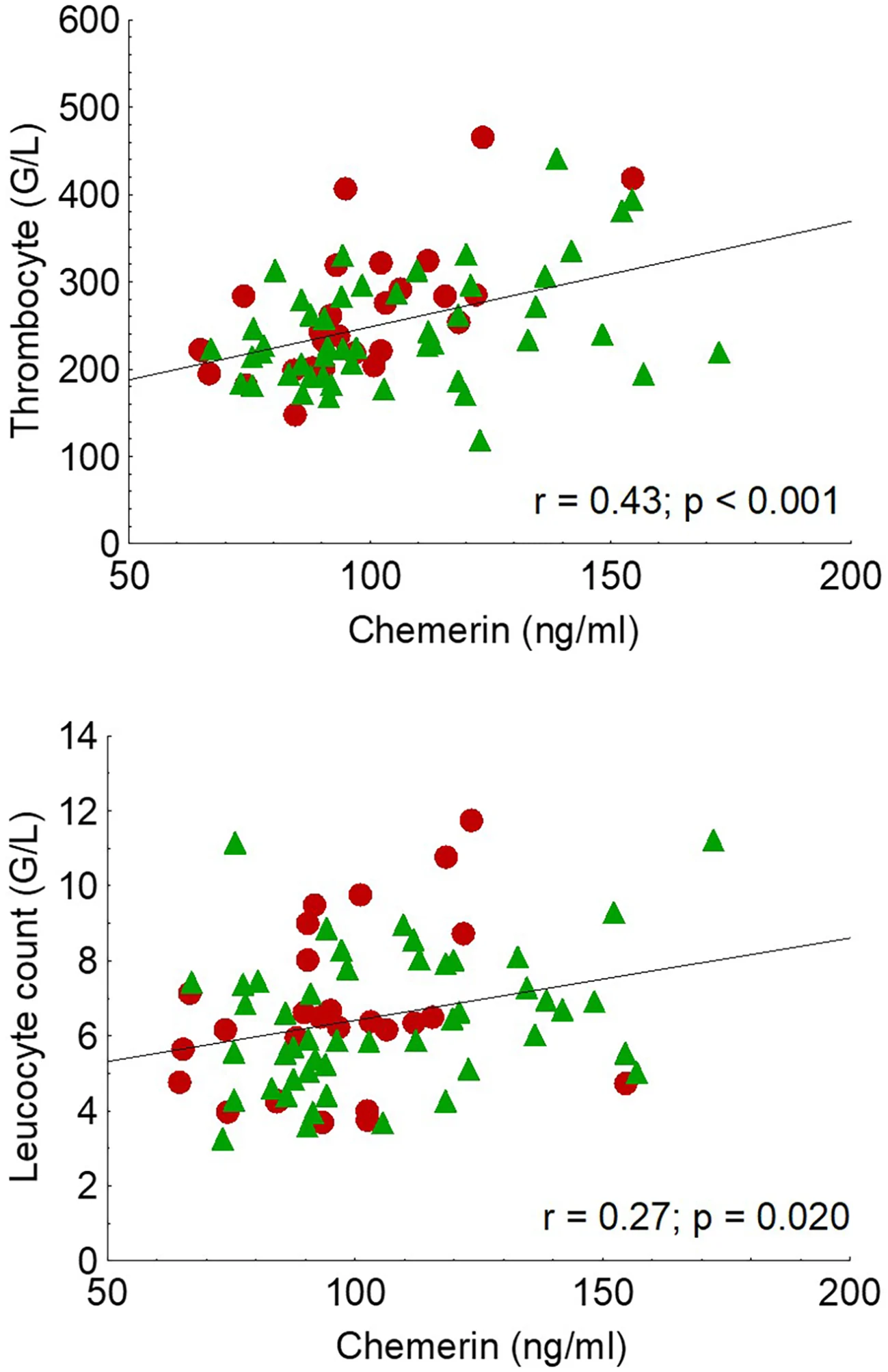
Correlation between platelet count and chemerin levels (green: overweight/obese, red: non-obese). Correlation between Leucocyte count and chemerin levels (green: overweight/obese, red: non-obese).

### 3.5 Platelet-associated inflammatory signaling as a determinant of chemerin levels

Backward stepwise multiple regression analyses were performed to determine significant predictor(s) of chemerin levels in overall, normal-weight and overweight/obese Sjögren’s disease cohort. Models were adjusted for BMI, gender, and age at the time of Sjögren’s diagnosis, and included all variables that correlated with chemerin in the univariate analyses (**Table 4**). In overall and in the obese group, BMI (overall: βstandardized=0.364; P<0.001 and overweight/obese: βstandardized=0.335; P<0.001) and platelet count (overall: βstandardized=0.524; P<0.001 and overweight/obese: βstandardized=0.456; P = 0.001) were independent predictors of chemerin. In normal-weight Sjögren’s patients, platelet count was a predictor of chemerin (βstandardized=0.0.647; P = 0.001).

**Table 4:** Backward stepwise multiple regression analyses to determine significant variables predicted chemerin in overall, overweight/obese and normal-weight Sjögren cohort.

Variable	Overall		Overweight/ obese		Non-obese	
	β_standardized_	P-value	β_standardized_	P-value	β_standardized_	P-value
BMI (kg/m^2^)	**0.364**	**<0.001**	**0.335**	**0.012**	0.181	0.248
Gender (m/f)	-0.073	0.455	-0.070	0.613	-0.174	0.269
Age at diagnosis (years)	0.137	0.164	0.110	0.429	0.098	0.610
Statin use (yes/no)	-0.043	0.670	-0.118	0.385	0.151	0.413
c3 (g/L)	0.041	0.748	0.160	0.226	-0.240	0.275
c4 (g/L)	0.138	0.192	0.111	0.522	0.184	0.377
log10hsCRP	-0.032	0.785	-0.124	0.402	0.144	0.424
oxLDL (U/L)	0.051	0.613	0.128	0.343	-0.219	0.295
**Platelet count (G/L)**	**0.523**	**<0.001**	**0.456**	**0.002**	**0.647**	**<0.001**
Leukocyte count (G/L)	0.073	0.481	0.115	0.416	0.044	0.845

Bold values indicate statistically significant results in the tables.

## 4 Discussion

According to our results, several parameters seem to be more favourable in overweight/obese patients, even the disease activity index (ESSDAI) is significantly lower than the patients with normal body weight.

Based on currently available academic information, we expected chemerin and oxLDL levels to be higher in the overweight/obese group, given their established associations with adipose tissue and metabolic dysfunction. However, regarding our patients, there was no significant difference between the two groups. Chemerin is also stated to be a pro-inflammatory adipokine, therefore, less beneficial parameters of the overweight/obese group were expected. The absence of a significant difference between the groups therefore suggests that the relationship between adiposity and circulating chemerin may be more complex in Sjögren’s disease.

In our cohort, mean chemerin levels in the overweight/obese group were lower, compared to results of several other available studies (36–39). Of course, having other types of kits used to determine chemerin levels could be one of the reasons behind this finding, but revising available data, there was no remarkable difference between results determined by different kits. However, we admit the limitations of our study, since it did not include a healthy control group and the difference between the overweight/obese and non-obese Sjögren’s groups was not statistically significant. The relatively lower chemerin concentrations observed in our overweight/obese patients may represent a potential hypothesis linking adipokine signaling with the lower ESSDAI observed in this group, but this possibility requires confirmation in larger, controlled and prospective studies.

As stated previously, chemerin plays an important role in the inflammatory process, mediating pathways and sustaining inflammation. According to several studies, chemerin is being researched as a potential biomarker for immunity and chronic diseases, as its elevated level could indicate the risk or severity of certain illnesses. Its biomarker aspect is already being researched in metabolic diseases, systemic lupus erythematosus, psoriasis vulgaris and rheumatoid arthritis. In psoriasis patients, a higher level of chemerin was found compared to healthy individuals and its level also correlated positively with the disease severity index. In Rheumatoid arthritis, high level of chemerin indicated higher clinical disease activity, more pronounced joint involvement and higher inflammatory marker levels (37, 39, 40). Considering these results, since chemerin levels of our overweight/obese patients were lower than expected, it is quite logical that their disease activity score indicates a less severe disease burden. In systemic lupus erythematosus, it did not prove to be a good marker of severity, however, a relevance was found between chemerin levels and specific organ involvements, such as lupus nephritis, as its circulating levels were found to correlate with kidney function in some studies (41).

One of the most notable findings of our study was the independent association between platelet count and circulating chemerin levels. Beyond their traditional role in hemostasis, platelets are increasingly recognized as active mediators of innate immunity and chronic inflammatory responses. Platelet activation contributes to endothelial dysfunction, leukocyte recruitment, cytokine release, and thromboinflammatory signaling, all of which are involved in autoimmune-mediated vascular injury and accelerated atherosclerosis. In autoimmune rheumatic diseases, elevated platelet count and platelet activation have already been associated with systemic inflammatory burden and vascular complications. Since chemerin also participates in inflammatory cell recruitment, adipose tissue signaling, and endothelial inflammatory pathways, the observed correlation may reflect a shared immunometabolic-inflammatory axis in Sjögren’s disease. This relationship could partly explain the link between systemic inflammation, adipokine dysregulation, and increased cardiovascular risk observed in these patients (42–44). Interestingly, platelet count remained an independent predictor of chemerin levels even after adjustment for BMI and demographic variables. This finding suggests that circulating chemerin concentrations may be influenced not only by adipose tissue mass but also by inflammatory and hematologic mechanisms. Considering the recognized role of platelets in endothelial activation and atherogenesis, platelet-associated inflammatory signaling may represent an additional pathway contributing to vascular dysfunction in Sjögren’s disease. Therefore, chemerin may represent a potential molecular interface between adipose tissue dysfunction, innate immune activation, and vascular inflammation in Sjögren’s disease.

As discussed in our previous paper, the significance of statin therapy must be emphasized. The substantially higher prevalence of statin therapy in the overweight/obese cohort represents a potentially important contributing factor. Given the pleiotropic anti-inflammatory effects of statins and their emerging interactions with adipokine signaling, part of the observed reduction in disease activity may reflect pharmacologic, rather than obesity-related immunomodulation, since it helps to reduce inflammation by interacting with pathways involving Rho/Rac signaling, NF-kB activation, elevating nitric oxide levels and lowering CRP level while also having immunomodulatory features (16–20). Statins can interfere with chemerin levels, however there is very limited available academic information about this, although, it could be explained by their anti-inflammatory effect. Some studies state that they can lower circulating chemerin levels by more than 30%, while PCSK-9 inhibitors did not seem to have any effect, making this a unique feature of statins (45).

Although oxLDL levels did not differ significantly between groups, platelet-associated inflammatory signaling appeared more strongly linked to chemerin levels, suggesting that cellular inflammatory pathways may be more relevant than circulating oxidized lipid burden in this cohort.

We acknowledge several limitations of our study. The relatively small sample size and single-center design may limit the statistical power and generalizability of our findings. Given that this was a cross-sectional study, causal relationships between adiposity, chemerin levels, inflammatory parameters, statin use, and disease activity cannot be established. Also, no healthy control group was included. Furthermore, our study was based primarily on clinically available data, and detailed lipid fractions and other potentially relevant metabolic and inflammatory parameters were not systematically assessed. The higher prevalence of statin therapy in the overweight/obese group represents an additional potential confounding factor, as statins may influence inflammatory and immunometabolic pathways, including chemerin signaling. Therefore, the observed differences between BMI groups cannot be attributed to adiposity alone. Finally, the available clinical literature on adipokines in autoimmune diseases remains relatively limited, making direct comparison and interpretation of our findings challenging. Larger, multicenter, controlled and prospective studies are warranted to validate these findings and further clarify the role of adipokines and platelet-associated inflammatory pathways in Sjögren’s disease. Nevertheless, adipokines represent a promising area of research, and we hope to further investigate their role and facilitate their integration into routine clinical practice.

## 5 Conclusions

In summary, our results suggest that overweight/obese patients with Sjögren’s disease may exhibit lower measured systemic disease activity despite the well-established adverse cardiovascular consequences associated with excess body weight. Distinct immune-metabolic and platelet-associated inflammatory pathways may contribute to disease activity and vascular risk in Sjögren’s disease. The greater use of statins among obese patients raises the possibility that statin-associated anti-inflammatory and immunometabolic effects may contribute to the observed differences, although this cannot be established in our cross-sectional study. Prospective studies should be carried out to investigate whether statin therapy influences chemerin levels, systemic inflammation, and disease activity in Sjögren’s disease. BMI and platelet count were independently associated with chemerin levels.

Nevertheless, this is the first paper assessing chemerin levels in patients with Sjögren’s disease.

## Data Availability

The raw data supporting the conclusions of this article will be made available by the authors, without undue reservation.
